# The Contribution of Dysfunctional Chloride Channels to Neurovascular Deficiency and Neurodegeneration

**DOI:** 10.3389/fphar.2021.754743

**Published:** 2021-10-04

**Authors:** David A. Gascoigne, Alexander Drobyshevsky, Daniil P. Aksenov

**Affiliations:** ^1^ Department of Radiology, NorthShore University HealthSystem, Evanston, IL, United States; ^2^ Department of Pediatrics, NorthShore University HealthSystem, Evanston, IL, United States; ^3^ Department of Anesthesiology, NorthShore University HealthSystem, Evanston, IL, United States

**Keywords:** GABA, hypoxia, development, anesthesia, interneurons

## Introduction

The brain is a metabolically demanding organ and its health directly depends on maintaining tissue oxygen that is sufficiently high to prevent hypoxia. Focal increases in oxygen demand, in response to sensory signals, motor output, etc., are supported by transient increases in cerebral blood flow via the hemodynamic response (Aksenov et al., 2016). Traditionally, specific products of glutamatergic and astrocytic pathways (i.e., nitric oxide (NO), arachidonic acid metabolites, calcium (Ca^2+^) and potassium (K^+^) ions) have been proposed as mechanistic contributors to the hemodynamic response (Archer et al., 1994; Attwell et al., 2010; Ross, 2012; Nippert et al., 2018). However, these mechanisms may not be sufficient drivers of the hemodynamic response. For example, a recent review (Nippert et al., 2018) concluded that, although NO must be present for vasodilation to occur in the cerebral cortex, it is not the active signaling molecule, arteriole vasodilation can occur in the absence of astrocyte Ca^2+^ increases, Ca^2+^ signals are characterized by long latencies occurring after the initiation of vasodilation and K^+^ siphoning through astrocytes does not always play a major role in neurovascular coupling. Moreover, hemodynamic modulatory pathways can have differing levels of influence across various structures. For instance, studies have shown that NO can be an active signaling molecule in the cerebellum (Akgoren et al., 1996; Yang and Iadecola 1997) and hippocampus (Lourenco et al., 2014).

A possible addition to this conventional approach are chloride channel-dependent mechanisms of neurovascular coupling, which may participate in neurovascular deficiency and neurodegeneration. Prominent pathways which employ such chloride channels are gamma aminobutyric acid (GABA) ergic interneuron pathways, which operate via GABA-gated chloride channels (GABA_A_ receptors) and provide a means of rapid signaling. The role of GABAergic interneurons and GABA_A_ receptors in inhibition of neuronal activity is well-known. Interneurons suppress excessive neuronal activity and spatially limit neuronal responses by instigating the hyperpolarization of the cell membrane which has the added benefit of decreasing local oxygen consumption. Additionally, GABA-gated chloride channels can directly participate in regulating cerebral blood flow. GABA_A_ receptors can be found along arterioles (Vaucher et al., 2000) where interneurons make direct morphological connections (Cauli et al., 2004; Tremblay et al., 2016). These chloride channels on brain vessels are functionally active and are able to facilitate substantial vasodilation in response to stimulation, attributable to the hyperpolarization of arteriolar smooth muscles with their subsequent relaxation. Multiple studies have shown that GABAergic interneurons are essential for the full expression of the hemodynamic response in the presence of chemical or electrical stimulation (Kocharyan et al., 2008), during epileptiform discharges (Saillet et al., 2016) as well as in response to both sensory (Aksenov et al., 2019) and optogenetic stimulation (Anenberg et al., 2015). Arteriolar GABA-gated chloride channels, can therefore play an important role in the hemodynamic response due to their fast and profound effect on vasodilation.

In essence, GABA-gated chloride channels can function to prevent hypoxia by both upregulating oxygen supply and downregulating oxygen consumption. Thus, it is our perspective that if the number of these channels or their main biochemical properties are affected, the combination of decreased inhibition and a weakened hemodynamic response can induce local hypoxia, which will alter the intracellular and extracellular environment with neurodegeneration evident thereafter. In support of this perspective, we will briefly review chloride channel dysfunction and neurodegeneration in different diseases, and then provide our interpretation regarding the role of neurovascular deficiency as a medium between chloride channel dysfunction and neurodegeneration.

## Neurodegeneration and Chloride Channel Deficiency

Chloride channel deficiency accompanies many neurodegenerative diseases. For example, in Alzheimer’s disease, which is characterized by progressive neurodegeneration starting in hippocampus and entorhinal cortex, the neurotransmission of GABA and GABAergic terminals have been shown to be significantly disrupted in areas neighboring beta-amyloid plaques (Li et al., 2016). Subsequent analysis has shown abnormal upregulation and downregulation of the α2, β1, γ1, and α1, γ2 subunits of GABA_A_ receptors respectively (Limon et al., 2012). Another example is Parkinson’s disease. This progressive neurodegenerative disorder is strongly associated with neuronal cell loss in the substantia nigra and striatum (Fahn and Sulzer, 2004). Although Parkinson’s disease mostly corresponds with the loss of dopaminergic neurons, GABA and GABA_A_ receptor deficiency has also been shown to play an important role in the early and non-motor symptoms of Parkinson’s disease (Murueta-Goyena et al., 2019). These changes in GABAergic pathways are different from those observed in Huntington’s disease. In Huntington’s disease GABAergic interneurons undergo specific morphological alterations (i.e., reduced somatic areas and dendritic field complexity) which accompanies aggressive neurodegeneration in the striatum (Bano et al., 2011).

The etiologies of Alzheimer’s, Parkinson’s and particularly Huntington’s diseases, are often attributed to genetics, however, some diseases (for example, epilepsy) can be independent of such substantial genetic factors. Distinctly, Drug-Resistant Epilepsy (DRE), which occurs in 40% of people with epilepsy (Engel, 2016), has been shown to cause neurodegeneration, often in the temporal lobe. Evidence has elucidated the association between the increased internalization of GABA_A_ receptors and symptoms in DRE (Goodkin et al., 2005; Naylor et al., 2005; Goodkin et al., 2007).

Even complex psychiatric disorders can present with chloride channel affiliated neurodegeneration. For instance, patients with schizophrenia exhibit progressive bilateral neurodegeneration in the grey matter of the temporal and parietal lobes (Whitford et al., 2006), and can exhibit significant under-expression of the α5 subunit of GABA_A_ receptors, the degree of which is correlated with the symptom severity (Marques et al., 2020). Furthermore, autism spectrum disorder (ASD) has demonstrated similar patterns of neurodegeneration to that of schizophrenia. Individuals with ASD have exhibited reduced grey matter volumes in the mirror neuron system (Hadjikhani et al., 2006; Marques et al., 2020). The severity of grey matter thinning in this area was further correlated with the severity of symptoms experienced by those with ASD. Moreover, genetic studies have identified copy number variations and entire locus duplications of the 15p11-q13 chromosomal region in patients with ASD, which lead to under and dysfunctional expression of the β3, α5, and γ3 subunits of GABA_A_ receptors (Hadjikhani et al., 2006). This indicates the potential of chloride channel deficiency to both precede cases of ASD, and have further downstream consequences of neurodegeneration.

Chloride channel dysfunction and neurodegeneration can also occur as an acquired iatrogenic condition; the most notable example of which is neonatal exposure to anesthesia (Aksenov et al., 2020a). Anesthetics that are classified as GABA agonists and glutamate antagonists (Aksenov et al., 2019), have consistently been shown to produce significant neuroapoptosis that is directly correlated with dosage and duration of the anesthesia delivery (Hadjikhani et al., 2006; Zheng et al., 2015; Liu et al., 2018). Moreover, the severity of apoptosis can create a loss of cortical neurons, of which a significant proportion are GABAergic inhibitory interneurons (Istaphanous et al., 2013), and a further study has shown general anesthesia to directly disturb chloride channels (Cabrera et al., 2020) thereby broadening the known contributory effects of anesthesia on neurodegeneration (Aksenov, 2021). These neurodegenerative and apoptotic processes can alter the delicate excitatory/inhibitory balance of cortical networks (Aksenov et al., 2020a). This imbalance can account for, at least in part, the negative developmental changes (Johnston et al., 2002; Aksenov et al., 2020a; Aksenov et al., 2020b) and impeded GABAergic system development (Young et al., 2012; Nisimov et al., 2018) following neonatal anesthesia. This disproportionate cell death leading to a shift in the excitatory/inhibitory balance requires further research in terms of occurrence of the local chronic hypoxia in later years, and how this shift caused by anesthesia, adapts throughout development.

## Discussion

We suggest that, in the absence of normal GABA_A_ receptor functioning, neurovascular deficiency could manifest where a weakened hemodynamic response, in combination with decreased inhibition, would be insufficient to support the present metabolic demand. Although this type of neurovascular deficiency does not result in actual ischemic stroke, it engenders chronic intermittent hypoxia which produces neurodegeneration. This clear sequence of events explains the importance of normal chloride channel functioning for preventing chronic hypoxia. Therefore, dysfunctional chloride channels could be a contributory factor to the neurodegeneration in the aforementioned diseases which are epiphenomenal with chloride channel dysfunction.

Indeed, the dangers of hypoxia on the intracellular and extracellular compositions of brain tissue have been well documented. It is known that insufficient oxygen for basic metabolic processes can lead to cell death (Mariotti et al., 2016). Although the neuronal damage is especially severe in sudden onset hypoxia–ischemia, such as in the case of an ischemic stroke, it can also occur as a result of chronic hypoxia (Dheer et al., 2018; Mahakizadeh et al., 2020). Depending on the severity, hypoxia has been shown to increase the production of reactive oxygen species which can accumulate beyond the protective abilities of anti-oxidative systems, causing oxidative stress (Chen et al., 2018). Oxidative stress has a high propensity to interact with macromolecules within cells (e.g., DNA/RNA oxidation, protein oxidation, nitration of tyrosine residues, and lipid peroxidation), leading to cell debilitation (Moreira et al., 2005). Other consequences of hypoxia include a reduction in intracellular and extracellular pH (Rolett et al., 2000; Yao and Haddad, 2004), phosphocreatine (Rolett et al., 2000), inorganic phosphate (Nioka et al., 1990; Rolett et al., 2000) and a buildup of NADH (Rolett et al., 2000; Shetty et al., 2014). These distinct alterations to the intracellular and extracellular environment significantly impair normal cellular functioning and have been shown to be biochemical indicators of neuroapoptosis. Such hypoxia-related events not only demonstrate the ability of insufficient cerebral blood flow to produce neurodegeneration in the immediate undersupplied tissues, but that it can also harmfully affect neighboring tissues as well.

Brain functioning and its metabolic support is a highly integrated process, and embedded within this complex system are GABAergic interneurons and the hemodynamic response. When neurodegeneration is present, determining if neurovascular deficiency precedes this process and exacerbates the neurodegeneration, or suffers as a direct consequence of an unbalanced excitatory/inhibitory system, remains a challenge. These two possibilities are accompanied by respective hypotheses and can therefore be examined by future studies in a controlled environment. A possibility of how one may address this issue includes *in vivo* studies providing longitudinal measurement of chloride channel and interneuron deficiencies in association with subsequent hemodynamic function and neurodegeneration.

Further interrogation into chloride channel subunit functioning may provide a bottom-up approach to more accurately describe their role in neurodegeneration. A family of genes have been identified (regions CLC2-7) to transcript chloride channels in the brain (Jentsch et al., 1999). These loci represent specific areas of potential genetic manipulation that could identify the discrete contribution of chloride channels and their subunits in degenerative diseases. In addition, the local modulation of chloride channel expression with a viral vector could be used. This type of methodology has proven effective in animal translational models (Miah et al., 2019). Unfortunately, little work has been done to use viral vectors to modulate chloride channel expression in the brain. However, in reference to GABA_A_ receptors, certain benzodiazepine derivatives have shown to allosterically bind to individual subunits. Namely, TPA023 (Atack et al., 2006), HZ166 (Di Lio et al., 2011) and SL651498 (Griebel et al., 2003) are reported to act as α2 and α3 agonists, while CGS 9865 binds to the β+α− interface (Maldifassi et al., 2016). Genetic and subunit-related research may provide further insights into chloride channel dysfunction and lead to etiologically-specific pharmacological solutions to both protect chloride channels, and prevent neurovascular deficiency, in the previously discussed diseases and conditions.
